# Diagnostic Performance of First Trimester Screening of Preeclampsia Based on Uterine Artery Pulsatility Index and Maternal Risk Factors in Routine Clinical Use

**DOI:** 10.3390/diagnostics10040182

**Published:** 2020-03-26

**Authors:** Max Mönckeberg, Valentina Arias, Rosario Fuenzalida, Santiago Álvarez, Victoria Toro, Andrés Calvo, Juan P. Kusanovic, Lara J. Monteiro, Manuel Schepeler, Jyh K. Nien, Jaime Martinez, Sebastián E. Illanes

**Affiliations:** 1 Department of Obstetrics and Gynecology, Faculty of Medicine, Universidad de Los Andes, Santiago 7620001, Chile; maxmonck@gmail.com (M.M.); mschepeler@gmail.com (M.S.); jyh.kae@gmail.com (J.K.N.); 2 Faculty of Medicine, Universidad de Los Andes, Santiago 7620001, Chile; vearias@miuandes.cl (V.A.); rdfuenzalida@miuandes.cl (R.F.); vvtoro@miuandes.cl (V.T.); adcalvo@miuandes.cl (A.C.); 3 Center for Research and Innovation in Maternal-Fetal Medicine (CIMAF), Hospital Sótero del Río, Santiago 8207257, Chile; jkusanovic@med.puc.cl; 4 Division of Obstetrics and Gynecology, School of Medicine, Pontificia Universidad Católica de Chile, Santiago 8331150, Chile; 5 Centre for Biomedical Research, Laboratory of Reproductive Biology, Faculty of Medicine, Universidad de Los Andes, Santiago 7620001, Chile; lmonteiro@uandes.cl; 6 Department of Obstetrics and Gynecology, Clínica Dávila, Santiago 8420384, Chile; jaimemartinez@movistar.cl

**Keywords:** preeclampsia, uterine artery Doppler, ultrasound, gestational hypertension, early prediction, aspirin, predictive algorithm, routine care, prenatal care

## Abstract

Preeclampsia is a pregnancy-specific disorder defined by new onset of hypertension and proteinuria after 20 weeks of gestation. The early detection of patients at risk of developing preeclampsia is crucial, however, predictive models are still controversial. We aim to evaluate the diagnostic performance of a predictive algorithm in the first trimester of pregnancy, in order to identify patients that will subsequently develop preeclampsia, and to study the effect of aspirin on reducing the rate of this complication in patients classified as high risk by this algorithm. A retrospective cohort including 1132 patients attending prenatal care at Clínica Dávila in Santiago, Chile, was conceived. The risk of developing preeclampsia (early and late onset) was calculated using algorithms previously described by Plasencia et al. Patients classified as high risk, in the first trimester of pregnancy, by these algorithms, were candidates to receive 100 mg/daily aspirin as prophylaxis at the discretion of the attending physician. The overall incidence of preeclampsia in this cohort was 3.5% (40/1132), and the model for early onset preeclampsia prediction detected 33% of patients with early onset preeclampsia. Among the 105 patients considered at high risk of developing preeclampsia, 56 received aspirin and 49 patients did not. Among those who received aspirin, 12% (7/56) developed preeclampsia, which is equal to the rate of preeclampsia (12% (6/49)) of those who did not receive this medication. Therefore, the diagnostic performance of an algorithm combining uterine artery Doppler and maternal factors in the first trimester predicted only one third of patients that developed preeclampsia. Among those considered at high risk for developing the disease using this algorithm, aspirin did not change the incidence of preeclampsia, however, this could be due either to the small study sample size or the type of the study, a retrospective, non-interventional cohort study.

## 1. Introduction

Preeclampsia (PE) and gestational hypertensive disorders (GHD) are amongst the most frequent medical conditions affecting pregnancy [1], with an estimated prevalence of approximately 2–10% [2,3,4]. These conditions represent one of the leading causes of maternal morbidity and mortality, and approximately 12–14% of maternal deaths occurring worldwide are attributed to these disorder [5,6]. PE is also associated with a significant increase in perinatal and neonatal morbidity and mortality, mainly due to preterm birth complications, intrauterine growth restriction and fetal death [7].

Given its epidemiological and clinical importance, several efforts have been made to predict and prevent the development of PE, using different predictive multiparametric algorithms based on maternal risk factors, uterine artery Doppler pulsatility index, and several plasmatic biomarkers with increasing effectiveness [8,9,10,11,12,13,14,15,16,17,18,19,20,21,22,23,24,25,26,27,28,29,30,31,32,33,34,35,36]. Nevertheless, actual predictive models for PE are still controversial and are not currently used in routine clinical care [2,37,38,39,40,41,42,43].

Since the 1980s, aspirin has been considered to play a role in the prevention of the disease [44,45,46,47,48,49]. A meta-analysis including 42 randomized trials and 27,200 pregnancies at high risk of PE demonstrated that prophylactic administration of aspirin, initiated before 16 weeks of gestation, was associated with a significant reduction of the risk of developing PE [relative risk(RR) = 0.47, 95% confidence interval (CI), 0.36–0.62), severe preeclampsia (sPE) (RR = 0.18, 95% CI, 0.08–0.41), perinatal mortality (RR = 0.41, 95% CI, 0.19–0.92) and intrauterine growth restriction (RR = 0.46, 95% CI, 0.33–0.64) [50]. Similar findings have been reported by several other systematic reviews and meta-analysis of randomized clinical trials [51,52,53,54,55,56].

In our clinical centre, we have organized a first trimester PE screening program based on the multi-parametric predictive model proposed by Plasencia et al. [57], considering maternal risk factors, biophysical characteristics and uterine artery Doppler velocimetry, which is offered to all our patients. The objective of this study was to perform a retrospective cohort study to evaluate the diagnostic performance of a first trimester screening program for PE in a general population setting, using the previously described combined predictive algorithm [57], and to evaluate the effects of aspirin administration in the incidence of PE when patients were classified as high risk using this predictive model.

## 2. Materials and Methods

### 2.1. Study Design and Settings

This is a retrospective cohort study including patients participating in the first trimester PE screening program of Clínica Dávila, a private tertiary healthcare centre located in Santiago, Chile.

### 2.2. Participants

Patients included in the first trimester PE screening program between January 2011 and June 2016 were eligible for the study. Informed consent was obtained from all individuals included in this study. This study has been complied with all the relevant national regulations, institutional policies and in accordance with the tenets of the Helsinki Declaration, and has been approved by the Institutional Review Board of Clínica Dávila, Chile. Inclusion criteria for the study were: 1) singleton pregnancies; 2) ultrasonographic evaluation performed between 11^0/7^–13^6/7^ weeks of gestation with crown-rump length between 45 and 84 mm; 3) confirmed fetal vitality; and 4) results of uterine artery pulsed Doppler velocimetry. Patients with a spontaneous abortion before 20 weeks of gestation, major fetal anomalies, missing data to calculate the risk for PE, and incomplete pregnancy follow-up were excluded from the study.

### 2.3. Procedures

First trimester ultrasound examinations were performed by experienced obstetricians, following the Fetal Medicine Foundation technical recommendations for the 11–14 weeks fetal scan. A General Electric Voluson 730 PRO ultrasound system was used. Uterine artery pulsed Doppler velocimetry was performed using a transabdominal or transvaginal probe, depending on physicians’ preference. The pulsatility index was measured in both uterine arteries and the average was recorded. Once patients fulfilled inclusion criteria, pregnancy medical records were reviewed. Maternal age, parity, obstetric history, medical comorbidities, use of anti-hypertensive drugs and aspirin during current pregnancy, as well as pregnancy and neonatal outcomes were recorded in a pre-established database. Attending physicians using locally certified instruments measured first trimester maternal weight, height and arterial blood pressure.

Using first the trimester ultrasound evaluation and the recorded maternal and obstetric data, all patients underwent an individual risk estimation of developing early- and late-onset PE (ePE and loPE, respectively) using the first trimester predictive algorithms described by Plasencia et al (2008). These two algorithms consist of a multiple logistic regression model that includes uterine artery pulsatility index [logarithm of pulsatility index expressed as multiples of the expected median (MoM)], personal history of chronic hypertension, parity, previous history of PE, family history of disease, and body mass index, to calculate an estimated individual risk of developing ePE and loPE. The second trimester ratio between uterine artery pulsatility index at 21^+0^–24^+6^ and 11^+0^–13^+6^ weeks described originally by Plascencia el al. (2008) [57] was not used for PE risk estimation in this cohort. Only the performance of the first trimester predictive algorithms was tested in the present study. Patients with clinical risk factors for developing PE, or those having a positive first trimester screening for PE, were candidates to receive aspirin 100 mg/day as prophylaxis. In this case, the indication of aspirin usage remained at the discretion of the attending physician. Pregnancies were followed up until delivery. Regular prenatal care was carried out by the attending physicians.

The primary outcome of the study was the development of PE, which was defined as new onset hypertension and proteinuria after 20 weeks of gestation according to recommendations of The American College of Obstetricians and Gynaecologists [2]. ePE was defined as delivery due to PE <34 weeks of gestation, and loPE when the delivery occurred >34 weeks.

### 2.4. Statistical Analysis

Qualitative variables are presented as absolute frequencies and percentages. Quantitative variables are presented as means and standard deviations. Comparisons of means between groups were performed using Student’s *t*-tests. Assumption of homogeneity of variances between groups was tested with Standard Deviation F-test, and an adjustment for *t*-test was performed in cases of inequality. Comparisons between proportions in qualitative data were performed with Pearson Chi-Square test or Fisher’s exact test as appropriate. Two-sided hypothesis tests were performed and *p*-values less than 0.05 were considered significant.

The individual estimated risk for developing early- and late-onset PE was calculated for each patient using the first trimester predictive models for both conditions previously described [57]. The 95^th^ centile of individual estimated risk was used for establishing the cut-off for the screening, thereby accepting a prefixed false positive rate of 5%. The diagnostic performance of the different screening algorithms was tested using detection rates, as well as positive and negative predictive values. Results are presented with 95% confidence intervals.

## 3. Results

After inclusion and exclusion criteria were applied, 1132 patients were considered for the study. The overall incidence of PE in the cohort was 3.5% (40/1,132). From those, 19 met the criteria for severe preeclampsia (1.7%). The mean gestational age at presentation of PE was 36.9 (±2.4) weeks. Six cases (0.5%) presented gestational age <34 weeks (ePE) and 34 cases (3%) presented gestational age >34 weeks (loPE). Maternal baseline characteristics at the first trimester assessment and pregnancy outcomes between affected and unaffected groups are presented in Table 1. Patients who subsequently developed PE had significantly higher values of systolic blood pressure and uterine artery pulsatility index in the first trimester than those who did not develop PE. Nulliparity and history of chronic hypertension were significantly associated with developing PE.

Based on the ePE predictive algorithm by Plasencia et al. [57], the mean estimated risk of ePE in our cohort was 1.01%. The 95th centile of estimated risk was 4.03%. Considering a fixed false positive rate of 5%, patients with an estimated risk over 4.03% were considered to have a positive screening for ePE. Based on loPE predictive algorithm, the mean calculated risk of loPE was 1.24% for the entire population. With a prefixed false positive rate of 5%, a calculated risk over 3.15% was considered a positive screening for loPE. Based on these calculations, 57 patients (5.04%) presented a positive screening for ePE, and 57 patients (5.04%) presented a positive screening for loPE.

The ePE predictive model detected 8 out of the 40 patients that developed PE in this cohort (20%), and two out of six cases of PE presenting <34 weeks of gestation (33%). Similarly, the loPE predictive model detected six (18%) of all PE observed and seven (18%) of 34 PE presenting after 34 weeks of gestation. Finally, when both predictive models were used in combination, 105 patients (9% of the cohort) tested positive for the screening. Through this combination, 33% of all PE were detected. Furthermore, 33% and 32% of ePE and loPE, respectively, were detected. The performance of the different screening models is summarized in Table 2.

The observed detection rates for ePE and loPE were compared with those originally reported by Plasencia et al. [57]. We observed no statistical significant differences between the detection rates observed in our study and those previously reported when a fix false positive rate of 5% was used (ePE observed detection rate: 33%, (2/6 cases), previously described: 46% (10/22 cases, Chi^2^=0.28, *p*-value=0.595); loPE observed detection rate: 32% (11/34 cases), previously described: 31% (22/71 cases, Chi^2^=0.02, *p*-value=0.888).

Among the entire cohort, 105 patients had a positive screening using ePE and loPE predictive models and were considered at high risk for developing PE. Amongst the patients testing positive for the combined screening in the first trimester, 56 received 100 mg/day aspirin and 49 patients did not, based on attending physician indications. In 53 out of the 56 high risk pregnancies who received prophylactic aspirin, its administration started before 16 weeks of gestation. Amongst the high-risk pregnant women whose aspirin administration started before 16 weeks of gestation (*n* = 53), six cases of PE were observed, whereas six cases were diagnosed between non-aspirin users (*n* = 49). In this group classified as high-risk, aspirin usage was not associated with a significant reduction in the risk of developing PE (RR: 0,91, 95% CI: 0.23–3.71, *p*-value = 0.88).

## 4. Discussion

This retrospective cohort study shows that in an unselected population, under routine clinical care, the use of a predictive model for PE based on uterine artery pulsatility index and maternal clinical and biophysical risk factors, with a fixed false positive rate of 5%, identified approximately one third of patients that will subsequently develop PE during their pregnancies, including one third of both ePE and loPE. The original study that generated the model used here described detection rates of PE (at a fixed false positive rate of 5%) of 46% for ePE and 31% for loPE [57]. There were no significant differences in the diagnostic performance between the original model presented by Plasencia et al. (2008), and the results obtained in the present study. These findings confirm the diagnostic capability of the models, even in an uncontrolled clinical scenario. Farina and colleagues also tested this predictive models in a prospective cohort study of 554 pregnancies screened for PE in the first trimester [58]. The authors observed a similar diagnostic performance of the models, with an overall detection rate of preeclampsia of 41% when using a false positive rate of 10%, therefore reaffirming our results and the external validity of the original predictive models used in these trials.

Although the detection rates observed in our study and of those reported before using the same models are similar, they are still very low considering its implementation in a routine clinical care. Indeed, the models did not identify almost 70% of patients that subsequently developed ePE, as well as nearly 70% of all cases of loPE. It is worth mentioning that in those previous studies, uterine artery PI were measured by transabdominal Doppler ultrasound, while in our study the use of transabdominal or transvaginal ultrasound was at the discretion of the operators. This is important, since it has been demonstrated that transabdominal and transvaginal Doppler ultrasound measurements of the uterine artery PI are significantly different, with the latter yielding significantly higher values than the former approach [59]. These methodological issues could explain the relatively lower detection rates observed in our study, compared to the aforementioned studies. However, given the similarities between the performance of the algorithm in our hands and what have been previously reported, it is unlikely that the Doppler method used is the cause of the overall poor performance of the test. Also, it is important to consider the possibility that the poor screening performance could be associated to a poor implementation that is due to the type of study, retrospective study, and that a stricter protocol could result in improvements of the screening performance.

When this screening method was used to select high-risk patients for the use of aspirin, no effect was observed in reducing the risk of developing PE. These findings suggest that performing a screening based only on uterine artery Doppler and maternal risk factors is insufficient to detect patients at risk of developing PE, especially those that would manifest the symptoms early in pregnancy and that would benefit more from the usage of aspirin. Nevertheless, the results observed regarding the role of aspirin administration for PE prevention must be taken with caution, due to several reasons. Firstly, the study sample size may be too low to demonstrate a benefit from aspirin in the high-risk pregnant women. Indeed, in the present study, 40 cases of PE were observed, including only 6 cases of ePE and 13 cases of preterm PE debuting under 37^+0^ weeks. Therefore, the vast majority of PE cases in our cohort presented at term. Moreover, recent evidence regarding the use of aspirin for PE prevention suggests that the benefits of this therapy are mainly for the prevention of preterm PE, with little or no effect in reducing the risk of PE at term [60,61]. Secondly, several methodological issues related to the design of the present study must be taken into account. This is a retrospective, non-interventional cohort study, in which the main objective was to test the performance of a PE screening program in general population under routine obstetrical care, therefore, its conclusions regarding therapeutic interventions should not be overestimated. The indication of aspirin therapy in high risk pregnancies remained totally dependent on the attending physicians, with no intervention of the researchers. In fact, almost half of patients with a positive screening did not receive aspirin despite the results of the screening program. Also, neither the clinicians nor the researchers took any action to ensure the compliance of patients regarding aspirin use. These methodological issues, which imply a high risk of selection and confounding biases, the relatively low capability of the screening models to correctly detect high risk pregnancies, and the small sample size of this research must be taken into consideration before drawing conclusions regarding the benefits of aspirin use.

Previous studies have suggested that the use of uterine artery velocimetry indexes alone (PI or RI over 95th centile) during first trimester screening of PE in non-selected obstetric populations has limited diagnostic performance, with sensibilities ranging between 21–27%, and positive and negative predictive values around 5–11% and 97–99%, respectively [23,62,63,64,65]. These diagnostic performances described in the literature suggest that early prediction of PE, using only uterine artery Doppler velocimetry, is insufficient for being applied in routine clinical care, and probably un-useful to decide whose patients would benefit from the use of aspirin.

Following these initial findings, in the last 10 years, more than 30 multiparametric predictive models, combining different maternal clinical risk factors with biophysical, biochemical and ultrasonographic markers, and using different statistical methodologies, have been developed for the screening of PE in early pregnancy, with a wide variation in terms of predictive capabilities [11,13,15,18,22,23,28,32,34,35,36]. Most recently combined predictive models, using maternal risk factors, serum angiogenic factors and other biomarkers, as well as uterine artery Doppler velocimetry and biophysical parameters, have consistently reached detection rates of 68–80% for predicting the development of PE <37 weeks, and over 85–90% for PE <34 weeks, considering a false positive rate of 10% [66,67,68].

Aspirin administration before 16 weeks of gestation has become the most important strategy for preventing PE [69,70,71,72]; therefore, the need for an effective first trimester screening model with clinical relevance is critical. Recently, Rolnik et al. [67], reported a multicentre randomized clinical trial (ASPRE Trial) in which 1776 patients at high risk of developing PE were randomized to receive 150 mg of aspirin or placebo from the first trimester. This trial reported a significant reduction in the incidence of preterm PE (<37 weeks) in the intervention arm (OR: 0.38, 95% CI, 0.20–0.74). In this study, patients were considered at high risk of developing PE using a prospective first trimester multi-parametric predictive model of PE based on maternal risk factors, uterine artery pulsatility index, mean arterial blood pressure, maternal serum pregnancy-associated plasma protein-A (PAPP-A), and placental growth factor (PlGF) concentrations. The detection rates observed in the trial were approximately 77% and 43% for early PE (ePE) and late onset PE (loPE), respectively, with a fixed false-positive rate of 10%. Similar results were obtained in other studies using the same combination of risk factors and biomarkers [20,66,73,74,75,76].

Despite the important advances in PE screening and prevention, the implementation of a first trimester screening program of PE still has several difficulties, especially in low-income countries. Maternal biomarkers, particularly PlGF and PAPP-A, are usually unavailable for routine clinical use, given its high costs and a lack of laboratories performing these tests. In addition, uterine artery Doppler is not universally available due to the lack of adequately trained ultrasound specialists. The low availability of first trimester screening for PE in low-income countries is particularly important considering that maternal mortality caused by hypertensive disorders in those countries is especially high [1,6,77,78,79,80,81].

The improvements in early predictive models for PE have allowed the selection of high-risk patients for whom early administration of aspirin has demonstrated effects on reducing the risk of developing PE. However, to obtain these results, proper early predictive models, at reasonable costs, should be implemented and tested in the routine clinical setting.

## Figures and Tables

**Table 1:** Maternal baseline characteristics, medical and obstetric history, and pregnancy outcomes in unaffected and preeclampsia groups.

	Unaffected (*n* = 1,092)	Preeclampsia (*n* = 40)	*p*-value
Maternal age (years)	30.6 (± 5.3)	30.3 (± 4.8)	0.776
Nulliparity	481 (44.1)	27 (67.5)	0.003
Crown-rump length (mm)	68.4 (± 9.0)	65.7 (± 9.0)	0.061
BMI (kg/m^2^)	26.4 (± 4.3)	26.3 (± 4.7)	0.905
History of preeclampsia	17 (1.6)	2 (5.0)	0.143
Systolic blood pressure (mmHg)	108 (± 10.2)	113 (± 12.7)	0.023
Diastolic blood pressure (mmHg)	65.4 (± 7.5)	65.9 (± 7.8)	0.680
Chronic hypertension	29 (2.7)	4 (10.0)	0.026
Pregestational diabetes mellitus	17 (1.6)	2 (5.0)	0.143
Mean uterine artery pulpability index	1.48 (± 0.5)	1.72 (± 0.6)	0.010
Aspirin use	96 (8.8)	12 (30.0)	<0.001
Gestational age at delivery (weeks)	38.3 (± 2.2)	37.0 (± 2.2)	0.001
Birthweight (grams)	3.358 (± 484)	3.039 (± 577)	<0.001

Values are mean (± standard deviation) or *n* (%). BMI: body mass index.

**Table 2:** Diagnostic performance of different screening models of preeclampsia in the first trimester of pregnancy, using a fixed false positive rate of 5% (*n* = 1132).

		Preeclampsia (*n* = 40)	Early-onset Preeclampsia (*n* = 6)	Late-onset Preeclampsia (*n* = 34)
Early-onset preeclampsia model	Detection rate: *n* (%):	8 (20)	2 (33)	6 (18)
	PPV (IC 95%):	14.04 (12.0116.06)	3.51 (2.44–4.58)	10.53 (8.74–12.31)
	NPV (IC 95%):	97.02 (96.03–98.01)	99.63 (99.27–99.98)	97.40 (96.47–98.32)
Late-onset preeclampsia model	Detection rate: *n* (%):	7 (18)	1 (17)	6 (18)
	PPV (IC 95%):	12.28 (10.37–14.19)	1.75 (0.99–2.52)	10.53 (8.74–12.31)
	NPV (IC 95%):	96.93 (95.93–97.94)	99.53 (99.14–99.93)	97.40 (96.47–98.32)
Combined models	Detection rate: *n* (%):	13 (33)	2 (33)	11 (32)
	PPV (IC 95%):	12.38 (10.46–14-30)	1.90 (1.11–2.70)	10.48 (8.69–12.26)
	NPV (IC 95%):	97.37 (96.44–98.30)	99.61 (99.25–99.97)	97.76 (96.90–98.62)

PPV: positive predictive value. NPV: negative predictive value.
